# Impact of Babaco (*Vasoncelea x pentagona* (Heilborn) Mabb.) Fruit Ripening Stages on Phytochemical Composition and Biological Properties

**DOI:** 10.3390/metabo14120718

**Published:** 2024-12-21

**Authors:** Raluca A. Mihai, Mauricio G. Canchignia Guacollantes, Ramiro F. Vivanco Gonzaga, Nelson S. Cubi-Insuaste, Rodica D. Catana

**Affiliations:** 1 Army Scientific and Technological Research Center—CICTE, Department of Life Science and Agriculture, Universidad de Las Fuerzas Armadas—ESPE, Av. General Ruminahui s/n y, Sangolqui 171103, Ecuador; gmcanchignia@espe.edu.ec (M.G.C.G.); rfvivanco2@espe.edu.ec (R.F.V.G.); nscubi@espe.edu.ec (N.S.C.-I.); 2 Developmental Biology Department, Institute of Biology Bucharest of Romanian Academy, 296 Splaiul Independentei, 060031 Bucharest, Romania; rodica.catana@ibiol.ro

**Keywords:** antioxidant activity, babaco fruit, phenolic compounds, maturity stages, nutraceuticals

## Abstract

**Background:** This research article delves into Babaco fruit’s—an Ecuadorian product with immense nutraceutical potential phytochemical composition and biological activity—in different maturation fruit stages. Bridging the gap between food and medicine, nutraceuticals offer health benefits beyond basic nutrition. **Methods:** Specifically, this study investigates Babaco’s antioxidant and its phenolic and flavonoid content across different ripening stages: physiological maturity, organoleptic quality immaturity, and commercial maturity. **Results:** This last stage of maturity exhibits the highest antioxidant activity, making it the optimal phase for marketing Babaco as a nutraceutical product. Further LC-MS analysis reveals the specific secondary metabolites responsible for this potent antioxidant capacity. **Conclusions:** By introducing Babaco as a nutraceutical fruit, Ecuador can showcase its unique phytochemical profile, which is rich in phenolic compounds and flavonoids. Consumers stand to gain from Babaco’s antioxidant properties, supporting overall health and well-being. Recognizing Babaco’s antioxidant potential aligns perfectly with Ecuador’s diverse biodiversity and offers a promising avenue for health-conscious choices.

## 1. Introduction

Nowadays, produce consumption has steadily climbed, paralleled by an exponential surge in demand for exotic crops. Consumers now recognize the nutritional value and health benefits inherent in these fruits, vegetables, and horticultural treasures. Notably, exotic fruits serve as more than mere culinary delights—they emerge as functional foods, rich in phytochemicals that underpin their health-promoting properties [1].

Antioxidants, found naturally in fruits, are crucial in reducing the oxidative stress in organisms. The pharmacological significance of antioxidants from natural sources lies in their beneficial effects on human health such as inhibiting various diseases—such as heart disease and cancer—preventing the free radicals’ negative impact, and protecting against the breakdown of nutrients and the benefits that have brought significant attention to antioxidants [2].

Ecuador, characterized by various climatic gradients from polar to tropical, is famous for its exceptional biodiversity, especially in the diversity of plants, many of which are used as food and traditional medicine [3]. Among these important crops is babaco (*Vasoncelea x pentagona*), a hybrid between distinct *Vasconcellea* species [4], a fruit-bearing shrub widespread in the Andean region, including Peru, and thriving in the native southern reaches of Ecuador. It belongs to the *Caricaceae* family, which comprises six genera and forty-two species, being similar to papaya (*C. papaya*). The babaco plant grows at high altitudes, between ~1200 and 2200 m above sea level, on highly fertile volcanic soil. The ideal conditions for its growth are temperatures between 14 and 27 °C, limited dampness, and prolonged sunlight. It favors sandy or clay soil and does not tolerate high salt concentrations [5]. Babaco fruit is 20–30 cm, weighs up to 1.5 kilos, has a pentagon-like shape, and has a smooth surface that turns bright yellow when fully mature. The pulp is white, watery, and tastes like a pineapple, strawberry, and papaya blend. Understanding the babaco fruit is crucial due to its significant economic sustainability and cultural heritage in Ecuador, preserving traditional agricultural practices. The production of Babaco provides an important source of income for numerous families in the country. Babaco, also called mountain papaya, holds cultural significance and conventional commercial applications (food, medicine, etc.) and culinary applications due to its unique taste and nutritional benefits [6].

**Figure 1:**
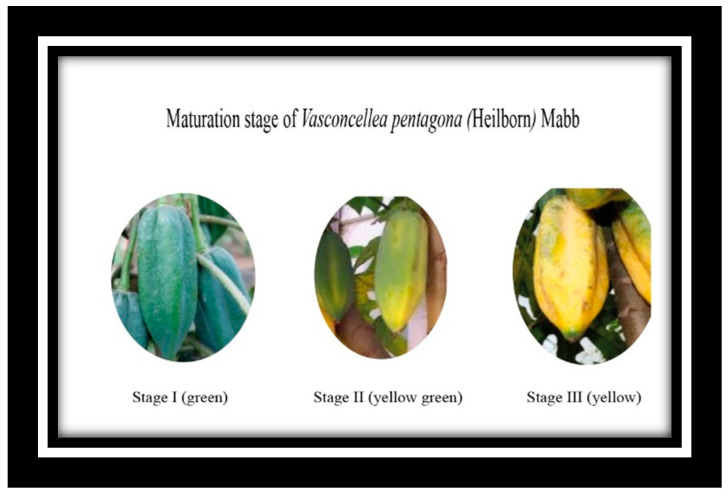
Different maturation stages of *Vasconcellea x pentagona*.

The antioxidant activity of this fruit, influenced by secondary metabolites (phenolic acids, flavonoids, carotenoids), varies in different ripening stages. This stage determines a fundamental characteristic for the consumption of any fruit: its antioxidant properties, which provide added value and drive its commercialization in the food industry [7]. To determine the stage of ripeness of the fruit, maturity indices based on its organoleptic quality, and sensory, and visual characteristics are used. These indices include a physiological state (Phase 1: unripe fruit, with dark color, no scent, strong firmness), incomplete ripeness or organoleptic maturity (Phase 2: pre-commercial pale-yellow fruit with also green color, low odor), and commercial maturity (Phase 3: yellow color, low firmness, strong odor) (Figure 1). Studying these variations can determine the optimal time for consumption to ensure the highest levels of beneficial compounds, which underlines the potential to promote health and prevent disease [6].

The legend is as follows: stage I—unripe fruit; stage II—pre-commercial pale-yellow fruit; stage III—commercial maturity.

For this reason, our research focuses on understanding the babaco fruit’s biological properties and antioxidant characteristics, which is essential for maximizing its nutraceutical benefits.

## 2. Materials and Methods

### 2.1. Sample Collection

Three babaco fruits were obtained at different stages of maturation: physiological maturity, organoleptic quality immaturity, and commercial maturity. The selection of the fruits was based on color as a qualitative criterion to determine their maturity index. The samples were collected in the parish of Guasaganda, Cotopaxi province.

### 2.2. Extract Preparation

After washing thoroughly with distilled water to remove any surface contaminants, the fruits were peeled, and the pulp was carefully extracted using a clean knife and spoon. The extracted pulp was homogenized using a food processor to create a uniform consistency. This step was essential to ensure even drying and maximize the exposure of the pulp to the drying environment. To prevent sticking, the homogenized pulp was spread evenly onto trays lined with parchment paper. The babaco pulp was freeze-dried to enhance the preservation of flavonoids and other phytochemicals. The samples were pre-frozen at −40 °C for 24 h and then subjected to freeze-drying under vacuum conditions (approximately 0.2 mbar) for 48 h. After drying, the samples were maintained at room temperature in a desiccator to prevent moisture absorption. The final weight of the dried pulp was measured using an analytical balance to determine the dry weight (g DW). The dried pulp samples obtained were stored in airtight containers at −20 °C until further analysis to prevent degradation of the bioactive components. From the total pulp, 5 g was taken for the subsequent extraction of bioactive compounds. The 5 g of pulp was rinsed with distilled water and strained to ensure that the sample was as fresh and pure as possible before proceeding with the extraction process.

### 2.3. Extraction of Bioactive Compounds

The extraction of bioactive compounds was performed following the protocol proposed by Correa [8], using the previously obtained babaco samples. The extracts were prepared by mixing 5 g of pulp with 50 mL of 99.5% ethanol. The resulting solutions were shaken (30 min) and centrifuged (5000× *g* rpm, 20 min). Subsequently, the supernatant was collected, discarded, and kept at −4 °C, for 72 h for supplementary analysis. Any remaining residues were discarded. This step ensures that only the pure extract is preserved for subsequent tests, enhancing the accuracy and reliability of the analysis of bioactive compounds. Pure extracts were used in all the tests, with no need for dilution.

### 2.4. Active Ingredients Determination

The quantification of phenolic content was based on the Folin–Ciocalteu method [9]. Solutions were prepared from the ethanolic extracts (0.4 mL) with 2 mL of Folin–Ciocalteu reagent (diluted to 10% *v*/*v*) and 1.6 mL of 7.5% Na_2_CO_3_, the mixtures being kept for 30 min at room temperature. Quantification was carried out by measuring the absorbance at 765 nm. The calibration curve was generated using gallic acid in a concentration range of 0 to 250 mg/L, yielding the equation y = 0.1399x + 0.1139, with a determination coefficient of R^2^ = 0.9737.

On the other hand, a colorimetric method based on the formation of complexes with aluminum chloride, as described by Pekal et al. [10], was used to determine the flavonoid content. A standard (quercetin) was used for the calibration curve, which was prepared in a concentration range of 0 to 1.5 mg/L, resulting in the equation y = 1.4566x + 0.0265, with a correlation coefficient of R^2^ = 0.9935. For the analysis, 1 mL of the crude extracts was blended with 1.5 mL of solvent, 100 µL of CH_3_COONa (1 M), 100 µL of AlCl_3_ (10% *v*/*v*), and 2.3 mL of distilled water. The samples were allowed to rest for 40 min at room temperature. In the case of blank samples, no aluminum chloride was added. The absorbance at 435 nm was measured to determine the flavonoid content.

### 2.5. Antioxidant Capacity Determination

To evaluate the antioxidant capacity through the ferric-reducing antioxidant power (FRAP) assay, a FRAP solution was prepared following the methodology described by Rajurkar et al. [11], using an acetate buffer solution (300 mM, 100 mL, pH 3.6), 40 mM HCl, and 20 mM FeCl_3_·6 H_2_O in 100:10:10. The absorbance used to measure the samples was 593 nm. The calibration curve was generated using various concentrations of FeSO_4_ × 7H_2_O (0–5 mM). For the analysis, 100 µL of the sample was blended with 300 µL of distilled water and 3 mL of the prepared FRAP solution and stood stand at room temperature for 30 min. The FRAP value (mmol Fe^2^⁺/g of sample) was calculated based on the difference between the sample absorbance and the control absorbance.

A protocol based on the work of Sachett et al. [12] and adapted by Thaweesang [9] was employed to evaluate the antioxidant capacity using the α-diphenyl-α-picrylhydrazyl free radical-scavenging (DPPH) radical. For this, a stock solution of 1 µg L⁻^1^ was prepared, from which 2 mL was added to each test tube, along with 0.1 mL of the crude extract sample. The samples were then incubated for 30 min at room temperature, after which absorbance was measured at 517 nm. The radical scavenging activity was calculated as the difference between the control absorbance and the sample absorbance divided by the control absorbance multiplied by 100. Trolox solution (0–0.625 mM) was used as a positive control, to create the calibration curve (R^2^ = 0.9824).

For the determination of the antioxidant capacity via free radical-scavenging activity (ABTS*^+^), free radical scavenging, the approach described by Kuskoski et al. [13] was followed. ABTS*^+^ radical was obtained through the reaction between ABTS (7 mM) and potassium persulfate, and the mixture was incubated for 12 to 24 h. The obtained solution was diluted with absolute ethanol until an absorbance of 0.7 ± 0.1 at 754 nm was achieved. The inhibitory capacity was assessed after the reaction between 2 mL of the ABTS solution and 20 µL of the sample for 7 min at room temperature in darkness, using absolute ethanol as a blank. The calibration curve (y = 34.102x + 9.2946, R^2^ = 0.9612) was established for concentrations between 0 and 2.5 mM, resulting in the equation using Trolox as a positive control.

### 2.6. Screening of Bioactive Compounds by LC-MS

To determine the bioactive compounds in the babaco fruits, liquid chromatography coupled with mass spectrometry (LC-MS) was used, following a modified method based on Tohma et al. [14]. Bioactive compounds were characterized via LC-MS exclusively at the third ripening stage of Babaco, corresponding to the commercial maturity stage. This stage was selected based on the premise that, at this point, the fruit exhibited its maximum bioactive profile, optimizing the detection of compounds with antioxidant properties. The extracts were achieved from lyophilized samples (1 g) using 20 mL of 80% ethanol, maintaining them at 30 °C for 2 h, and centrifuging at 5000× *g* rpm for 10 min at 4 °C. After this, the extracts were filtered, and ethanol was removed using a rotary evaporator at 30 °C. The samples were stored at −20 °C in sealed plastic tubes until analysis.

The metabolites were identified and detected using an HPLC system (Vanquish, Thermo Fisher Scientific, Waltham, MA, USA) and a mass detector (Ion Trap, Thermo Fisher Scientific, Waltham, MA, USA ). The samples were eluted through an Accucore Vanquish 150 × 2.1 mm column at 35 °C, with a flow rate of 0.5 mL/min [15]. A 10 μL volume of 0.1% formic acid was used as the mobile phase for injection into the HPLC. Compound identification was carried out through mass scanning, comparing the retention time of each peak and the ions monitored in a standard solution Tohma et al. [14]. The compounds were identified by comparing them with the reference compounds available in databases (PubChem, ChEBI, Metlin, HPLC) and literature data, using MZmine 2.53 software [16].

### 2.7. Statistical Test

The statistical analysis was conducted using RStudio software (version R 4.3.2). Significant differences between the groups were evaluated using a two-way ANOVA, with a significance level of *p* < 0.05. All the experiments were performed 3 times and the results were expressed as the mean ± standard deviation (SD). The correlation between the analyzed secondary metabolites and antioxidant capacity was determined using a post hoc Tukey test. The data were visualized in bar graphs with error bars, utilizing the ggplot2 package to clearly and precisely represent data variations.

## 3. Results

### 3.1. Active Ingredients Determination

**Figure 2:**
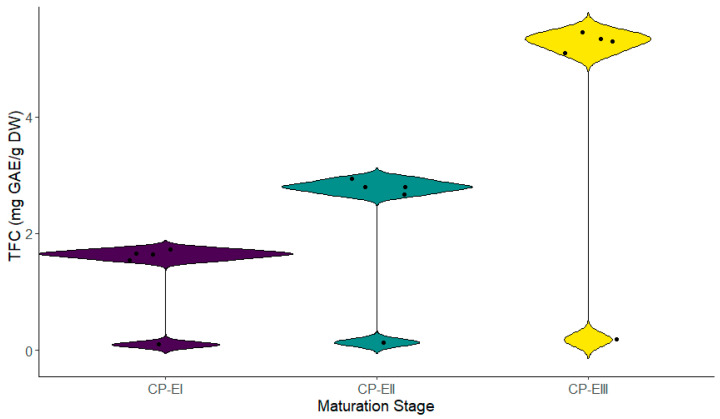
Total flavonoid content at different stages of fruit maturation. Legend: stages CP-E I, II, and III correspond to the different phases of the fruit’s maturation cycle.

**Figure 3:**
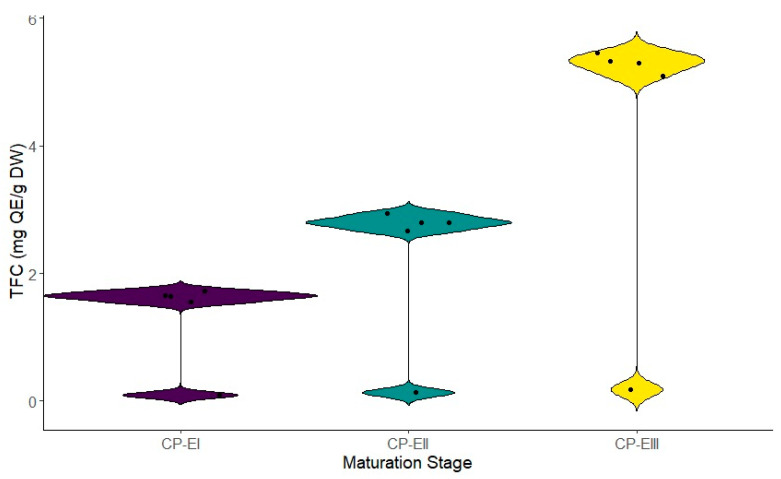
Total phenolic content at different stages of fruit maturation. Legend: stages CP-E I, II, and III correspond to the different phases of the fruit’s maturation cycle.

The active ingredients represented by the total flavonoid (TFC) and phenolic content (TPC) were evaluated at each stage of fruit maturation. Significant variations throughout the stages were observed. Stage III exhibited the highest flavonoid content (5.294 ± 0.179 mg GAE g DW), followed by Stage II (2.801 ± 0.131 mg GAE g DW) and Stage I (1.641 ± 0.093 mg GAE g DW) (Figure 2). The same pattern was noted for phenolic content (Figure 3). These results suggest a progressive accumulation of these compounds as the fruit develops, which could be related to an increase in the antioxidant defense mechanisms in the later stages.

### 3.2. Evaluation of the Antioxidant Capacity

**Figure 4:**
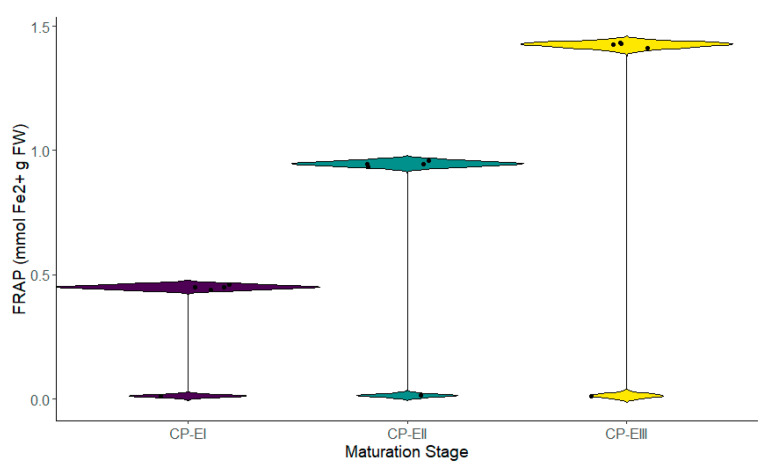
Ferric reducing antioxidant power at different stages of fruit maturation. Legend: stages CP-E I, II, and III correspond to the different phases of the fruit’s maturation cycle.

**Figure 5:**
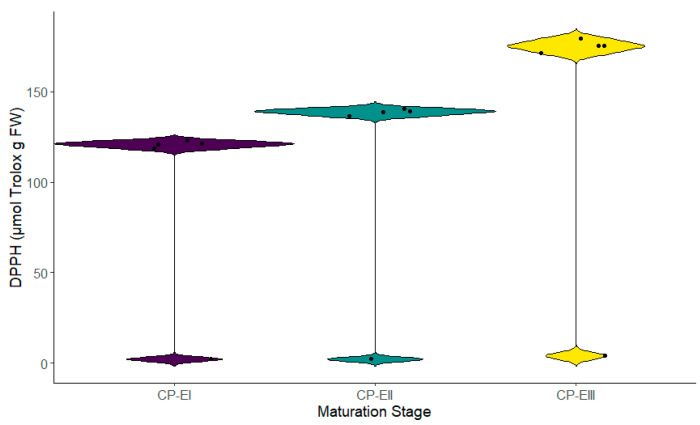
Antioxidant capacity measured by DPPH radical at different stages of fruit maturation. Legend: stages CP-E I, II, and III correspond to the different phases of the fruit’s maturation cycle.

**Figure 6:**
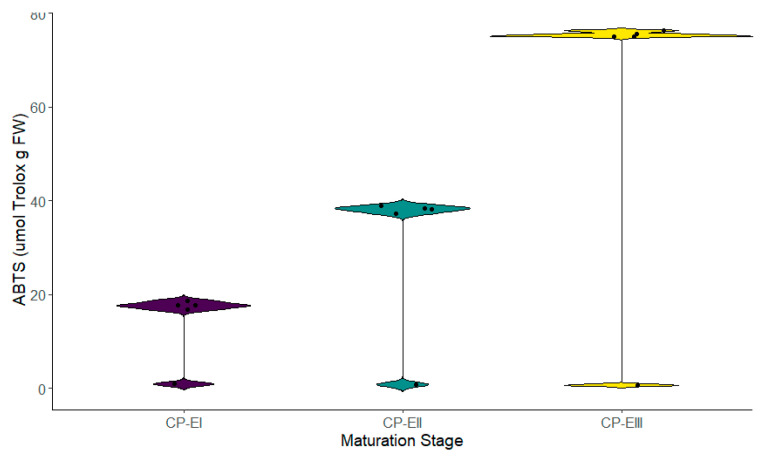
Antioxidant capacity by ABTS* + free radical scavenging at different stages of fruit maturation. Legend: stages CP-E I, II, and III correspond to the different phases of the fruit’s maturation cycle.

The antioxidant activity in the different stages of fruit maturation was evaluated through three different methods, showing significant differences across its cycle. Stage III recorded the highest reducing capacity (1.425 ± 0.012 mmol Fe^2^⁺ g dry weight—DW), followed by Stage II (0.945 ± 0.013 mmol Fe^2^⁺ g DW), and Stage I (0.451 ± 0.011 mmol Fe^2^⁺ g DW) (Figure 4). The same tendency was observed in the case of the antioxidant activity determined through the DPPH radical (Stage III—175.216 ± 2.061 µmol Trolox g fresh weight (FW), followed by Stage II with 138.802 ± 2.061 µmol Trolox g FW and Stage I with 120.845 ± 3.891 µmol Trolox g FW), and the ABTS*+ radical (Stage III—75.513 ± 0.681 µmol Trolox g FW, Stage—38.227 ± 0.816 µmol Trolox g FW, and Stage I—17.705 ± 0.921 µmol Trolox g FW) (Figure 5 and Figure 6). These results, like TPC and TFC, suggest a progressive increase in the antioxidant capacity as the fruit advanced in its development.

**Figure 7:**
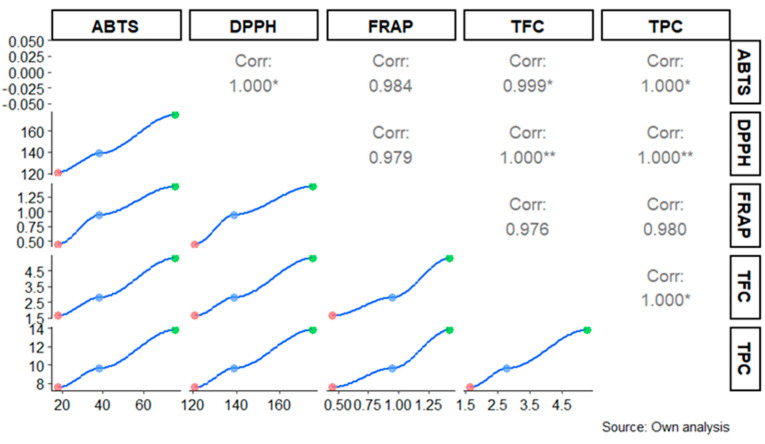
Correlation between flavonoid and total phenolic content with ABTS, DPPH, and FRAP assays at different maturation stages of *Vasconcellea x pentagona* fruit. Correlation values are expressed by the maturation stage and are classified as ** medium correlation, * low correlation. Different color dots indicate the data points for each maturation stage, and the lines represent the fitted correlation trend for the respective assays. Legend: FRAP—Ferric Reducing Antioxidant Power Assay, DPPH—DPPH assay, ABTS*+—Free Radical Scavenging Assay.

The results showed that the total phenolic and flavonoid contents exhibited a high positive correlation (R > 0.90) with the three methods used to assess antioxidant capacity (ABTS, DPPH, and FRAP) at each maturation stage. This finding suggests that, regardless of the maturation stage, there is a strong and consistent relationship between the presence of these bioactive compounds and the measured antioxidant capacity in the fruit (Figure 7).

### 3.3. Screening of Bioactive Compounds by Liquid Chromatography Coupled with Mass Spectrometry LC-MS

LC-MS was employed for the identification of bioactive compounds in the babaco samples. The analysis, based on molecular mass and retention times, revealed a range of significant compounds. Several bioactive compounds were identified through LC-MS positive ions and another 20 through LC-MS negative ions. The compounds identified include 20% organic acids, 12.5% flavonoids and phenolic compounds, and 7.5% amino acids and saponins (Table 1). Key bioactive molecules identified include honokiol, known for its potential antioxidant and anti-inflammatory properties. Other notable compounds such as urolithin B, a metabolite of ellagitannins with potential health benefits, and zeatin-9-glucoside, a cytokinin glucoside, were also detected. Flavonoids like rutin and rosmarinic acid, recognized for their ability to combat oxidative stress, were identified as well. The presence of amino acids such as arginine, along with other relevant compounds, underscores the biochemical complexity of babaco. These results suggest that the antioxidant capacity of babaco may result from the synergistic interaction of multiple metabolites. Consequently, these findings highlight the potential of babaco fruit as a rich source of natural antioxidants.

## 4. Discussion

The relentless pursuit of humans in deriving benefits from their environment has been characteristic since their early days on our planet. Plants, particularly fruits, are no exception to this study due to their dietary benefits and functions as natural preservatives [17]. The presence of endogenous antioxidant molecules in most fruits has been a topic of study in recent years due to their potential to prevent diseases and serve as natural substitutes for synthetic antioxidants [18].

Starting from the moment of fruit formation by the plant, it acquires nutrients synthesized by the leaves, providing beneficial molecules for its development, including vitamin C, vitamin A, Fe, Ca, and P, as well as bio-active compounds such as phenols, amino acids, saponins, tannins, and flavonoids [19]. The presence of these bioactive compounds in the fruit generates additional interest due to their direct relationship with the antioxidant capacity they can generate and provide [20].

As fruits develop, they go through various ripening stages, during which they complete their development and show higher amounts of phytochemicals thanks to various internal chemical reactions that generate powerful antioxidant properties [21].

In addition to considering the overall quality of babaco fruit, it is crucial to recognize other properties that make its consumption highly recommended, particularly its functional values such as antioxidant activity. While there have been a few quality studies focusing on babaco at the time of harvest, there is a notable lack of research on the fruit’s ripening stages and their influence on its functional properties. Understanding these aspects can provide valuable insights into optimizing the nutraceutical benefits of Babaco.

Our results are in the same trend pattern as Domínguez [22], demonstrating that as the pineapple cv. “Esmeralda” fruit matures, the phenolic compounds and antioxidant capacity increase, with the highest in the fourth ripening stage. Also, Aryal et al. [23] pointed out that the total content of these compounds was directly related to the antioxidant capacity. The increase is explained by the increased hydrolysis of tannins, which promotes the increase in phenol content, thereby enhancing the antioxidant character [24].

The fruits of this genus contain high percentages of dietary fiber and compounds with high reducing power, including bioactive compounds such as polyphenols, vitamin C, and carotenoids [25]. Different factors can determine the presence or absence of these bioactive compounds including ripening stages, the cultivation location, the extraction solvent, etc. [26]. For this study, absolute ethanol (EtOH) was used as a solvent for samples from the Andean region (Guasuganda-Cotopaxi) at three post-harvest ripening stages. The use of ethanol to obtain these extracts, was due to its ability to dissolve the endogenous compounds present in fruit (peels and pulp) [26]. In the case of phenolic compounds, according to Sultana et al. [27], they have a hydroxyl group that facilitates their high solubility in polar organic solvents, achieving greater efficacy when aqueous solutions of the solvent are used rather than in its absolute form. The use of ethanol or methanol as solvents can vary the results numerically but not conceptually. However, due to methanol’s higher polarity compared to ethanol, it is recommended to use the former solvent to avoid significant biases in the results obtained [8].

Results using the ABTS method showed the highest radical inhibition values in the third stage (CP-EIII) with average inhibition values of 73.673%, corresponding to the antioxidant activity of 75.51 µmol Trolox/g FW, and for DPPH, 175.22 µmol Trolox/g FW. A study by Auquiñivin and Paucar (2020) [28] quantified antioxidant activities using the ABTS and DPPH methods in *Carica pubescens* (mountain papaya) and *Vasoncelea x pentagona*, obtaining values of 37.67 ± 1.54 and 12.61 ± 0.61 µmol Trolox/g FW for ABTS, and 149.23 ± 8.01 µmol Trolox/g FW and 127.51 ± 4.23 µmol Trolox/g FW for DPPH. This study did not reveal the ripening stage at which the samples were collected. Still, the values were similar to those quantified in this work for the first ripening stage, with average values of 17.70 µmol Trolox/g FW for ABTS and 120.85 µmol Trolox/g FW for DPPH, suggesting that the samples collected by Auquiñivin and Paucar [28] were at physiological maturity due to the value coincidence in the same species of babaco.

Using the DPPH method to determine antioxidant capacity, the highest radical inhibition values were observed in the samples at the third ripening stage (CP-EIII) with values of 80.392%, compared to the less mature samples (CP-EI = 55.826% and CP-EII = 64.939%). Mejía [29] calculated the % inhibition of the pulp samples from *Vasoncelea x pentagona* at the green and mature ripening stages, measuring the antioxidant activity as % radical inhibition scavenging using methanol as a solvent. The study found a significantly higher % inhibition in the mature stage than the green stage, with values of 79.02 ± 3.44% and 60 ± 1.29%, respectively. In the same study by Mejía [29], the total phenolic content was measured, yielding results of 10.39 ± 0.19 mg GAE/g FW for the mature stage and 7.26 ± 0.49 mg GAE/g FW for the green stage. Our research showed numerically different but conceptually similar results for the total phenolic content (CP-EI = 7.547; CP-EII = 9.633; CP-EIII = 13.831 mg GAE/g FW). Oniszczuk et al. [30] indicate that the relationship between antioxidant capacity and total phenolic content is directly proportional, with higher antioxidant activity depending on the hydroxyl groups in the molecule. The aglycones in the structure generate greater antioxidant activity than glycosidic forms.

Quantitative studies to determine the total flavonoid content for the type of babaco used in this research have not yet been developed. Most studies summarize these as phenolic compounds or refer only to total phenols. However, Zunjar et al. [31] conducted a study on *Carica papaya* in India, quantifying the total flavonoid content using LCMS-MS, with 22.99 ± 0.02 µg QE/mg values. For babaco fruit, the highest flavonoid quantities were quantified at the third ripening stage, with a value of 5.29 mg QE/g FW, indicating that although both species belong to the same genus, results vary depending on the geographical location of the sample and the method used to quantify flavonoids.

For the FRAP method, Amagua [32] determined, in his study on diseased babaco leaves under the effect of elicitors (salicylic acid, methyl jasmonate), the inhibition of the Fe^2+^ complex with an average antioxidant capacity of 104.322 and 100.663 µM of Fe/g, respectively, obtaining the best results in the plants treated with salicylic acid. Our data shows the results obtained with a higher Fe^2+^ reduction capacity for babaco fruit at its third ripening stage, with an average value of 1.425 mM Fe^2+^.

The correlation values between the total phenolic and flavonoid content shown in Figure 6 evidenced high correlations between the antioxidant capacity and total phenols (ABTS-TPC, R^2^ = 0.97; DPPH-TPC, R^2^ = 1.00; FRAP-TPC, R^2^ = 0.99) and the total flavonoids (ABTS-TPC, R^2^ = 1.00; DPPH-TPC, R^2^ = 0.95; FRAP-TPC, R^2^ = 0.99). According to Lino et al. [33], phenolic and flavonoid compounds donate hydrogen atoms to free radicals, promoting their deactivation, providing significant antioxidant activity, and being structurally suitable for eliminating free radicals.

This study demonstrated the existence of bioactive principles, such as polyphenols, that substantially contribute to the antioxidant capacity and reduce the power of babaco pulp, depending on the ripening stage. There is a positive association connecting the amount of phenolic and flavonoid compounds and antioxidant activity, regardless of the ripening stage. However, as the fruit ripens, there is a varying presence of phenols and flavonoids, resulting in varying antioxidant activity.

Different active compounds were identified in the babaco fruit in their commercial maturity (Phase 3) like mefenamate (shown efficacy in treating pain and inflammation), urolithin B, a metabolite of ellagitannins, which has shown potential benefits like anti-inflammatory and antioxidant effects. Urolithin B has also been studied for its role in muscle health and longevity [34]. Zeatin-9-glucoside, a cytokinin glucoside, is involved in plant growth regulation and has been researched for its potential anti-aging effects in humans [35]. Further analysis revealed the presence of arginine, an amino acid important in protein synthesis and nitric oxide production, which plays a crucial role in cardiovascular health; 11-Aminoundecanoic acid, a compound with potential applications in polymer synthesis, is used in the production of nylon-11, a bioplastic [36]. Additionally, the detection of pterosin B, a compound with potential anti-cancer properties, and aloin A, known for its laxative effects and potential skin benefits, emphasizes the complex bioactive profile of the fruit [37]. Also, in our sample, we identified ureidopropionic acid, a metabolite involved in the metabolism of uracil, with implications for nucleotide metabolism [38]. Among the bioactive compounds evaluated in babaco, rutin (C_27_H_30_O_16_) and rosmarinic acid (C_18_H_16_O_8_) were identified as the primary chemical markers associated with their antioxidant capacity. Rutin, a well-studied flavonoid, is notable for its high activity in scavenging free radicals and contributing to the antioxidant capacity of plant systems [39]. Rosmarinic acid, meanwhile, is a phenol with antioxidant and anti-inflammatory properties that complement rutin’s activity [40]. These compounds were detected in babaco samples using liquid chromatography-mass spectrometry (LC-MS), evidencing a significant bioactive profile across the ripening stages analyzed. The accumulation of rutin and rosmarinic acid in babaco suggests that its antioxidant capacity may largely be attributed to the presence of these flavonoids. This finding highlights the importance of these compounds as indicators of the antioxidant quality in fruit, which will be essential for future research into the functional benefits of *Vasoncelea x pentagona*. The detection of these compounds at the commercial maturity stage underscores Babaco’s potential for nutraceutical applications and suggests that this ripening stage is crucial to harnessing its functional properties.

## 5. Conclusions

This study on babaco fruit samples at three different post-harvest ripening stages (physiological, incomplete ripening, and commercial ripening) revealed significant findings regarding their bioactive compound content and antioxidant activities. The results demonstrated that the babaco samples at the third ripening stage exhibited the highest concentrations of total phenols, averaging 13.83 mg GAE/g FW, and of total flavonoids, averaging 5.29 mg QE/g FW. These samples also showed the highest antioxidant activities, with average inhibition values of 75.51 µmol Trolox/g FW for the ABTS assay and 175.22 µmol Trolox/g FW for the DPPH assay. Additionally, the reducing power quantified by the FRAP method was highest in the third-stage samples, with an average value of 1.425 mM Fe^2+,^ corresponding to an activity of 712.70 mg FeSO_4_/100 g FW.

These findings indicate a directly proportional relationship between the antioxidant activity and the total content of the phenols and flavonoids in the babaco fruit samples. Therefore, the third ripening stage of babaco is optimal for maximizing its antioxidant properties, highlighting the importance of consuming the fruit at this stage to obtain the greatest health benefits.

Moreover, this research highlights the importance of traditional and local crops in contributing to health and nutrition. The high antioxidant activity observed in babaco fruit underscores its potential as a functional food with significant health benefits. Promoting the consumption of babaco, particularly at its peak ripening stage, could enhance dietary antioxidant intake and offer protective health effects. In conclusion, this study not only provides valuable insights into the bioactive properties of babaco but also emphasizes the critical role of proper harvesting and consumption timing in optimizing the nutritional value of this unique fruit.

## Figures and Tables

**Table 1:** Screening of bioactive compounds of babaco fruits by LC-MS.

LC–MS Positive Ions				
ID	Proposed Compound Identity	Molecular Formula	Retention Time (Minutes)	Molecular Ion
27	Honokiol	C_18_H_18_O_2_	1.089	M + H
76	Urolithin B	C_13_H_8_O_3_	1.082	M + H
129	Zeatin-9-glucoside	C_14_H_21_N_5_O_6_	1.114	M + H
189	Arginine	C_6_H_14_N_4_O_2_	1.22	M + H
215	11-Aminoundecanoic acid	C_11_H_23_NO_2_	1.239	M + H
305	Pterosin B	C_14_H_10_O_3_	1.293	M + H
504	Aloin A	C_21_H_22_O_9_	1.503	M + H
548	Ureidopropionic acid	C_4_H_8_N_2_O_3_	1.54	M + H
640	Isoliquiritigenin	C_15_H_12_O_4_	1.58	M − H
870	(E)-Hexadec-7-enoylhomoserine	C_19_H_35_NO_4_	1.647	M + H
883	N-Acetyl-alpha-D-glucosamine	C_8_H_15_NO_6_	1.655	M + H
908	Rutin	C_27_H_30_O_16_	1.552	M + H
931	Hecogenin	C_27_H_42_O_4_	1.685	M + H
948	Rosmarinic acid	C_18_H_16_O_8_	1.783	M + H
1009	N-Acetylhistidine	C_8_H_13_N_3_O_3_	3.191	M+
1021	Isoliquiritin	C_21_H_22_O_9_	9.356	M + Na
**LC-MS Negative Ions**				
**ID**	**Proposed compound identity**	**Molecular Formula**	**Retention Time (minutes)**	**Molecular Ion**
5	2-Benzylsuccinic acid	C_11_H_12_O_4_	398	M − H
52	8-hydroxy-2,7,7,11,15-pentamethyl-5,12,16-trioxapentacyclo [9.8.0]nonadec-13(18)-ene-3,17-dione	C_20_H_28_O_3_	1114	M − H
93	2-Isopropylmalate	C_7_H_12_O_5_	1216	M − H
99	Glucose, fructose, mannose	C_6_H_12_O_6_	1.26	M − H
102	alpha,alpha-Trehalose	C1_2_H_22_O_11_	1238	M − H
154	Citric acid	C_6_H_8_O_7_	1516	M + H
270	Rutin	C_27_H_30_O_16_	1648	M − H
403	(3S)-8-hydroxy-3-methyl-3,4-dihydro-2H-benzo[a]anthracene-1,7,12-trione	C_19_H_14_O_4_	16,729	M − H
405	(S,E)-(2-(4,8-dimethylnona-3,7-dien-1-yl)-8-formyl-5-hydroxy-2-methyl-2H-chromen-7-yl)methyl acetate	C_21_H_28_O_4_	16,769	M – Ac – H -
440	Ginsenoside	C_42_H_72_O_14_	17,439	M + HCOO
441	2-hydroxy-4-methoxy-3,5-bis(3-methylbut-2-enyl)-6-(2-phenylethyl)benzoic acid	C_27_H_32_O_4_	17,442	M + H
456	8,15-DiHETE	C_20_H_32_O_4_	18,022	M − H
514	2′,6′-Dihydroxy-4-methoxychalcone-4′-O-neohesperid	C_23_H_24_O_11_	19,838	M + HCOO
570	5Alpha-Cholestan-3Beta-Ol-6-One	C_27_H_46_O_2_	21,764	M − H
595	1-(3,4-dihydroxyphenyl)-6,7-dihydroxy-1,2-dihydronaphthalene-2,3-dicarboxylic acid	C_18_H_12_O_6_	22,049	M + Na
629	(E)-5-[(1S,8aS)-5,5,8a-trimethyl-2-methylidene-3,4,4a,6,7,8-hexahydro-1H-naphthalen-1-yl]-3-(hydroxymethyl)-2-oxopent-3-enoic acid	C_20_H_28_O_3_	22,361	M + H
652	(Z)-5,8,11-trihydroxyoctadec-9-enoic acid	C_18_H_32_O_5_	22,704	M − H
696	Aphyllic Acid	C_16_H_18_O_2_	28.29	M + H
726	Hydroquinidine	C_20_H_26_N_2_O_2_	29,577	M + H

## Data Availability

The original contributions presented in this study are included in this article; further inquiries can be directed to the corresponding author due to privacy.

## References

[1] Kumar A.P.N., Kumar M., Jose A., Tomer V., Oz E., Proestos C., Zeng M., Elobeid T.K.S., Oz F. (2023). Major Phytochemicals: Recent Advances in Health Benefits and Extraction Method. Molecules.

[2] Rahaman M.M., Hossain R., Herrera-Bravo J., Islam M.T., Atolani O., Adeyemi O.S., Owolodun O.A., Kambizi L., Daştan S.D., Calina D. (2023). Natural antioxidants from some fruits, seeds, foods, natural products, and associated health benefits: An update. Food Sci. Nutr..

[3] Dafermos G.N., Vivero Pol J.L. (2014). The Open Agri-Food System 1 of Ecuador: A commons-based transition towards sustainability and equity to reach a Buen Vivir for all. Environ. Sci. Agric. Food Sci. Econ..

[4] Kyndt T., Romeijn-Peeters E., Van Droogenbroeck B., Romero-Motochi J.P., Gheysen G., Goetghebeur P. (2005). Species relationships in the genus *Vasconcellea* (Caricaceae) based on molecular and morphological evidence. Am. J. Bot..

[5] Repositorio Digital del Instituto Nacional de Investigaciones Agropecuarias (INIAP) EGuía para el Cultivo de Babaco en el Ecuador. https://repositorio.iniap.gob.ec/handle/41000/515.

[6] Aguirre-Rodríguez A., Duarte-Casar R., Rojas-Le-Fort M., Romero-Benavides J.C. (2024). Food uses, functional activities, and bioactive compounds of three Ecuadorian Vasconcellea fruits: Bibliometric analysis and review. J. Agric. Food Res..

[7] Buelvas-Caro S.D., Assia-Ortiz M.C., Polo-Corrales L. (2018). Non-thermal Treatments for Food Preservation. Indian J. Sci. Technol..

[8] Correa Y. Capacidad Antioxidante y Contenido de Compuestos Fenólicos del Extracto Hidroalcohólico de la Pulpa de *Vasconcellea x heilbornii* (Babaco). Universidad César Vallejo, Facultad De Ciencias De La Salud Escuela De Nutrición. 2016. Licenciada en Nutrición. https://repositorio.ucv.edu.pe/bitstream/handle/20.500.12692/72178/Correa_TY-SD.pdf?sequence=1&isAllowed=y.

[9] Thaweesang S. (2019). Antioxidant activity and total phenolic compounds of fresh and blanching banana blossom (Musa ABB CV. Kluai “Namwa”) in Thailand. IOP Conf. Series Mat. Sci. Eng..

[10] Pękal A., Pyrzynska K. (2014). Evaluation of aluminum complexation reaction for flavonoid content assay. Food Anal. Met..

[11] Rajurkar N.S., Hande S.M. (2011). Estimation of phytochemical content and antioxidant activity of some selected traditional Indian medicinal plants. Indian J. Pharm. Sci..

[12] Sachett A., Gallas-Lopes M., Conterato G.M.M., Herrmann A., Piato A. (2021). Antioxidant activity by DPPH assay: In vitro protocol. Protocols Io. https://www.protocols.io/view/antioxidant-activity-by-dpph-assay-in-vitro-protoc-btbpnimn.

[13] Kuskoski E.M., Asuero A.G., Troncoso A.M., Mancini-Filho J., Fett R. (2005). Aplicación de diversos métodos químicos para determinar actividad antioxidante en pulpa de frutos. Food Sci. Technol..

[14] Tohma H., Köksal E., Kılıç Ö., Alan Y., Yılmaz M.A., Gülçin İ., Bursal E., Alwasel S.H. (2016). RP-HPLC/MS/MS analysis of the phenolic compounds, antioxidant and antimicrobial activities of *Salvia* L. species. Antioxidants.

[15] Irakli M., Skendi A., Bouloumpasi E., Chatzopoulou P., Biliaderis C.G. (2021). LC-MS identification and quantification of phenolic compounds in solid residues from the essential oil industry. Antioxidants.

[16] Luskal T., Castillo S., Villar-Briones A., Orešič M. (2010). MZmine 2: Modular framework for processing, visualizing, and analyzing mass spectrometry-based molecular profile data. BMC Bioinform..

[17] Munekata P., Pateiro M., Domínguez-Valencia R., Nieto G., Kumar M., Dhama K., Lorenzo J.M. (2023). Bioactive Compounds from Fruits as Preservatives. Foods.

[18] Bisbal J.J.S., Lloret J.M., Lozano G.M., Fagoaga Garcia C. (2020). Especies vegetales como antioxidantes de alimentos (Plant species as food antioxidants). NEREIS Rev. Iberoam. Interdiscip. Métodos Model. Simulación.

[19] Oliveira M.B., Sales R.P., Pereira M.C.T., Mouco M.A.C., Ferreira J.D., Cano R.N., Kondo M.K., Santos I.P., Martins R.S., Pegoraro R.F. (2019). Maturation and quality of ‘Palmer’ and ‘Espada Vermelha’ mango fruits in the Brazilian semi-arid. Acta Hortic.

[20] Nemzer B.V., Kalita D., Yashin A.Y., Yashin Y.I. (2020). Bioactive Compounds, Antioxidant Activities, and Health Beneficial Effects of Selected Commercial Berry Fruits: A Review. J. Food Res..

[21] Oszmiański J., Lachowicz S., Gorzelany J., Matłok N. (2018). The effect of different maturity stages on phytochemical composition and antioxidant capacity of cranberry cultivars. Eur. Food Res. Technol..

[22] Domínguez C.R., Domínguez Avila J.A., Pareek S., Villegas Ochoa M.A., Ayala Zavala J.F., Yahia E., González-Aguilar G.A. (2018). Content of bioactive compounds and their contribution to antioxidant capacity during ripening of pineapple (*Ananas comosus* L.) cv. Esmeralda. J. Appl. Bot. Food Qual..

[23] Aryal S., Baniya M.K., Danekhu K., Kunwar P., Gurung R., Koirala N. (2019). Total Phenolic Content, Flavonoid Content and Antioxidant Potential of Wild Vegetables from Western Nepal. Plants.

[24] Muñoz R., de las Rivas B., López de Felipe F., Reverón I., Santamaría L., Esteban-Torres M., Curiel J.A., Rodríguez H., Landete J.M., Frias J., Cristina Martinez-Villaluenga C., Elena Peñas E. (2017). Chapter 4—Biotransformation of Phenolics by Lactobacillus plantarum in Fermented Foods. Fermented Foods in Health and Disease Prevention.

[25] Vega-Gálvez A., Poblete J., Rojas-Carmona R., Uribe E., Pastén A., Goñi M.G. (2021). Vacuum drying of Chilean papaya (*Vasconcellea pubescens*) fruit pulp: Effect of drying temperature on kinetics and quality parameters. J. Food Sci. Technol..

[26] Chen C., Mokhtar R.A.M., Sani M.S.A., Noor N.Q.I.M. (2022). The Effect of Maturity and Extraction Solvents on Bioactive Compounds and Antioxidant Activity of Mulberry (*Morus alba*) Fruits and Leaves. Molecules.

[27] Sultana B., Anwar F., Ashraf M. (2009). Effect of extraction solvent/technique on the antioxidant activity of selected medicinal plant extracts. Molecules.

[28] Auquinivin S., Aldo E., Menacho P., Luz M. (2020). Comparative study of the physicochemical characteristics and shelf lifeof native papayas, “monte papayita” (*Carica pubescens* Lenné & K. Koch) and “babaco” (*Carica pentagona* Heilborn) (Caricaceae) dehydrated by lyophilization. Arnaldoa.

[29] Mejia A., Fany B. Caracterización fisicoquímica de la pulpa de babaco (*Vasconcellea x heilbornii*) en dos estados de madurez procedente de tres lugares de la región Amazonas. Bachelor’sThesis, Universidad Nacional Toribio Rodríguez de Mendoza de AmazonasGraduate, Chachapoyas, Peru, 2022. https://hdl.handle.net/20.500.14077/2875.

[30] Oniszczuk A., Widelska G., Wójtowicz A., Oniszczuk T., Wojtunik-Kulesza K., Dib A., Matwijczuk A. (2019). Content of Phenolic Compounds and Antioxidant Activity of New Gluten-Free Pasta with the Addition of Chestnut Flour. Molecules.

[31] Zunjar V., Mammen D., Trivedi B.M. (2015). Antioxidant activities and phenolics profiling of different parts of *Carica papaya* by LCMS-MS. Nat. Prod. Res..

[32] Sucuzhanay C., Leon Zeas R.L., Patino F.X., Emperatriz V. (2010). Evaluación del comportamiento del babaco (Vasconcella x heilbornii nm.pentagona) en tres tipos de alturas de podas en plantas de seis años de producción en la Parroquia Bulán, cantón Paute, Provincia del Azuay. Bachelor’s Thesis.

[33] Lino F.M.A., de Sá L.Z., Torres I.M.S., Rocha M.L., Dinis T.C.P., Ghedini P.C., Somerset V.S., Gil E.S. (2014). Voltammetric and spectrometric determination of antioxidant capacity of selected wines. Electrochim. Acta.

[34] Chen P., Guo Z., Chen F., Wu Y., Zhou B. (2022). Recent Advances and Perspectives on the Health Benefits of Urolithin B, A Bioactive Natural Product Derived From Ellagitannins. Front. Pharmacol..

[35] Rattan S., Sodagam L. (2005). Gerontomodulatory and Youth-Preserving Effects of Zeatin on Human Skin Fibroblasts Undergoing Aging In Vitro. Rejuvenation Res..

[36] Martino L., Basilissi L., Hermes F., Ortenzi M., Zini E., Di Silvestro G., Scandola M. (2014). Bio-based polyamide 11: Synthesis, rheology and solid-state properties of star structures. Eur. Polym. J..

[37] Sánchez-Machado D.I., López-Cervantes J., Sendón R., Sanches-Silva A. (2017). Aloe vera: Ancient knowledge with new frontiers. Trends Food Sci..

[38] Diwan V., Brown L., Gobe G.C. (2017). The flavonoid rutin improves kidney and heart structure and function in an adenine-induced rat model of chronic kidney disease. J. Funct. Foods.

[39] Nijveldt R.J., van Nood E., van Hoorn D.E., Boelens P.G., van Norren K., van Leeuwen P.A. (2001). Flavonoids: A review of probable mechanisms of action and potential applications. Am. J. Clin. Nutr..

[40] Amoah S.K., Sandjo L.P., Kratz J.M., Biavatti M.W. (2016). Rosmarinic acid—Pharmaceutical and clinical aspects. Planta Medica.

